# Primordial Germ Cell Specification and Migration

**DOI:** 10.12688/f1000research.6995.1

**Published:** 2015-12-16

**Authors:** Florence Marlow

**Affiliations:** 1 Department of Developmental and Molecular Biology, Albert Einstein College of Medicine, Yeshiva University, Bronx, NY, 10461, USA; 2 Department of Neuroscience, Albert Einstein College of Medicine, Yeshiva University, Bronx, NY, 10461, USA

**Keywords:** Primordial Germ Cell, Primordial Germ Cell Specification, Migration, gametes, germ plasm, Germ Plasm Assemblers, Germ Granules, Germline Identity

## Abstract

Primordial germ cells are the progenitor cells that give rise to the gametes. In some animals, the germline is induced by zygotic transcription factors, whereas in others, primordial germ cell specification occurs via inheritance of maternally provided gene products known as germ plasm. Once specified, the primordial germ cells of some animals must acquire motility and migrate to the gonad in order to survive. In all animals examined, perinuclear structures called germ granules form within germ cells. This review focuses on some of the recent studies, conducted by several groups using diverse systems, from invertebrates to vertebrates, which have provided mechanistic insight into the molecular regulation of germ cell specification and migration.

## Introduction

**Figure 1.**
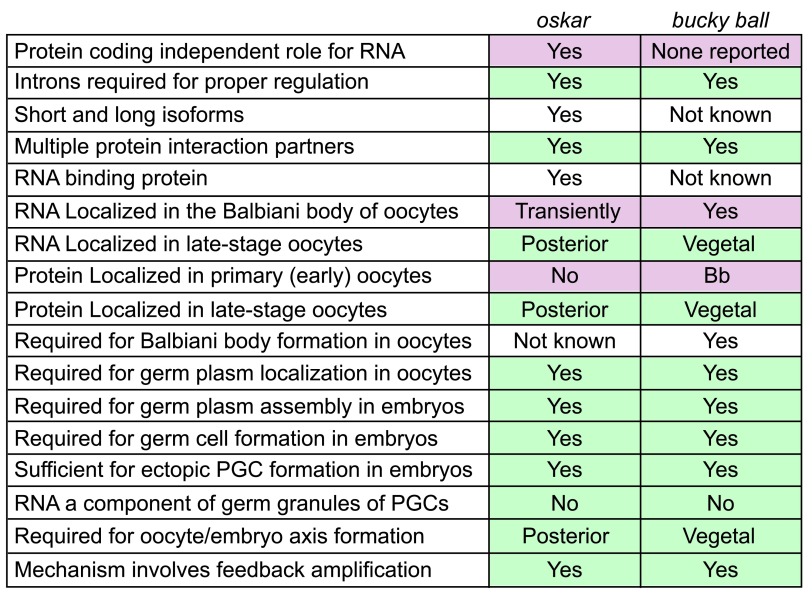
Comparison of two maternal germ plasm assemblers: *oskar* and *bucky ball*. The left column lists activities, localization, or other properties. Those attributed to *Drosophila oskar* or zebrafish *bucky ball* or both are indicated in the relevant columns. PGC, primordial germ cell.

In 1892, August Weismann challenged the notion that the germline (reproductive cells) was derived from the soma (cells of the body). Instead, Weismann proposed that the germ cells possessed a special immortal substance called “ancestral germ plasm” that was inherited from germ cells of one generation to the next in his “theory of the continuity of the germ plasm” ^1, 2^. Germ plasm is a maternally supplied substance comprised of RNAs, proteins, and organelles that are amassed in oocytes and later is sequestered during the first embryonic cleavages within a few cells that will become the primordial germ cells (PGCs). In the next century, the identification of conserved germline-specific markers and the germ cell accumulation of alkaline phosphatase made it possible to trace the origins of the germline from its earliest emergence through PGC migration to the presumptive gonad where they differentiate as male or female gametes. Such lineage tracing revealed that indeed some animals establish their germline by inheritance of maternal factors and post-transcriptional regulation in the context of a silenced genome but that others do not. In the latter case, these animals lack detectable maternal germ plasm and induce their germ cells by zygotic transcription factors. Genetic and overexpression screens to identify germ cell inducers have uncovered only a few factors with the capacity to generate ectopic PGCs. *Oskar* ^3^ and *bucky ball* ^4^ are “drivers” of germline fate among animals that use a maternal inheritance mode of PGC specification, but these genes are specific to different subsets of species ^5– 7^, and it remains to be determined whether their mechanisms of action and specific activities are conserved ( Figure 1). In humans, which use an inductive mode of specification, *sox17* is sufficient to specify human primordial germ cell-like cells (hPGCLCs) ^8^, but in mouse no germ cell inducer has been identified. This suggests that only a few genes possess germ cell-inducing activity or that the coordinated action of multiple genes is required to establish the germline or both. Consistent with this notion, many factors involved in germ cell development are involved in RNA regulation, including Vasa, a universal marker of and regulator of germ cell development ^9– 26^. Recently, investigators proposed a “last cell standing model”, whereby early PGC determination, as occurs in maternal germ plasm inducers, is not an innovation to protect germline traits. Instead, they proposed that germ plasm provides a means to specify the germline lineage earlier, before gastrulation, and thereby liberate the somatic cells of the embryo to more rapidly evolve ^27^. According to this model, innovation in animals that do not specify the germline early—prior to or in early gastrulation either by maternal germ plasm or inductive modes—but instead specify PGCs late in gastrulation by zygotic inductive modes are constrained by signaling and morphogenesis requirements associated with germ layer specification and gastrulation ^27^. Significantly, many of the same or related genes regulate PGC development independently of specification mode. This review focuses on the earliest events of PGC specification in the zygote, the mechanisms that induce early PGC-like cells in culture.

## Perinuclear accumulations in germ cells

Weismann recognized the germ plasm as a peculiar and complicated structure, but at that time the molecular components were not known. Since then, germ cell-specific accumulations have been detected in germ cells at all stages of the germline cycle. Further adding to the complexity, germ cell substances that have been identified throughout the germline cycle have been referred to by a variety of terms—nuage, germ plasm, and germ granules, or P-granules—at different developmental stages and in different animals. In recent years, molecular identification of the components of these germ cell substances has revealed that the molecular components overlap somewhat and thus nuage, germ plasm, and germ granules have at times been treated as largely equivalent. This assumption has made it challenging to decipher the functions of these germ cell manifestations, particularly when attempting to compare studies of these germ cell substances conducted at different developmental stages and in different organisms. To facilitate comparisons of functional studies between species, definitions of nuage, germ plasm, and germ granules in the context of this review are provided below.

Nuage is the perinuclear granulo-fibrillar electron-dense material that has been identified through histological and ultrastructural examination of oocytes from invertebrates through vertebrates and that is present in various conformations in cells of the male and female gametes ^28^. Although nuage occupies a distinct subcellular space, it is generally not asymmetric in its distribution. In some animals, a subset of nuage components become asymetrically localized to a specific subcellular location in oocytes where the germ plasm that is transmitted from oocytes to the embryo forms ( Figure 1). This substance is present prior to zygotic genome activation and contains germ cell-inducing activity ( Figure 1). After germ cell specification and zygotic genome activation, germline-specific aggregates of proteins and RNA-binding proteins called germ granules form next to the nucleus ( Figure 1). The functions of each of these germ cell-specific substances and their distinguishing features are active areas of study.

As an initial step toward defining nuage function, the identity of nuage molecules has been sought. The products of conserved germline-specific genes that are necessary for germ cell development, such as Vasa protein, are enriched in perinuclear regions where nuage is found ^16, 18, 29, 30^. Nuage has been postulated to be involved in the maternal germ plasm pathway; however, nuage is present even in animals that do not use inheritance of maternal germ plasm to specify their germline, indicating that nuage components may have other functions. It is also possible that nuage in animals that use inductive modes also contains germ plasm precursor materials, but that a nucleator or assembly/scaffold factor that can assemble nuage components into active germ plasm is lacking. Notably, evidence suggests that specification by zygotic induction is the ancestral mode of germline determination in insects and vertebrates ^6, 7, 29, 31^. Maternal specification by germ plasm in some insects has been associated with the presence of the germ plasm inducer *oskar*, discussed in the following section. Oskar is thought to have been co-opted from an ancestral neural role and to have facilitated the transition from zygotic germ cell induction to maternal specification via germ plasm ^6, 7^. Based on the conserved presence and perinuclear localization of nuage and the nature of the molecules that localize there, nuage functions in processes other than maternal germ plasm assembly have been proposed.

Among molecules that are enriched in nuage are proteins involved in genesis of piwi-interacting RNAs (piRNAs). piRNAs are components of a gonad-specific RNA silencing pathway that is thought to protect genome integrity by counteracting transposable or selfish genetic elements that promote their own transmission at the expense of other elements (reviewed in 32, 33). piRNAs are small RNAs that are produced from the cleavage of precursor RNAs by endonuclease activity of germline-specific members of the Argonaut family, called Piwi proteins, and secondary amplification ^34, 35^. Argonaut family members, including Piwi and Aubergine in flies, and human Argonaut homologs have been implicated in transcriptional and post-transcriptional regulation of gene expression ^36^. Like Vasa protein, Piwi protein homologs reside adjacent to the nucleus within the nuage in some species (reviewed in 37). Based on the conserved localization of piRNA pathway components as well as genetic and other functional data, a conserved nuage role as the site of piRNA amplification has been proposed ^38– 56^. While this may indeed be the case, not all piRNA components localize to the nuage (reviewed in 37). Moreover, the phenotypes of some piRNA pathway components suggest additional functions in diverse processes, including nuclear functions in chromosome rearrangements, chromosome dynamics, roles in RNA metabolism and storage, stem cell maintenance, regulation of cell divisions at stages before germ plasm would assemble in oocytes, or later roles in PGC maintenance; these roles have generated models whereby nuage and later germ granules serve to extend the nuclear environment and have been reviewed elsewhere ^57^. Owing to its dynamic nature, its varied composition at different stages, phenotypic differences between genders and species, and multiple stage-specific activities indicated by nuage component mutants, including piRNA pathway molecules, the developmental functions of nuage are not fully understood.

## Germ plasm assemblers

In primary oocytes of some animals, maternal germ plasm first assembles within an ancient perinuclear oocyte structure known as the Balbiani body ^28, 58, 59^ and later is found at the oocyte cortex, the posterior pole in some insects or the vegetal pole of some vertebrates ^28, 58, 59^ ( Figure 1). Expression-based screens have identified germ plasm components and candidate regulators on the basis of their localization to sites of germ plasm assembly in oocytes, such as the Balbiani body of early oocytes or the cortex of late-stage oocytes (reviewed in 15, 59, 60) *.* However, localization to the germ plasm is only suggestive of potential function as not all molecules that localize to germ plasm are essential for its assembly or activity ^16– 64^. Functional assays to define the component(s) of germ plasm that can impart germ cell identity, the germ plasm nucleators or assemblers, have included isolation and transplantation of cytoplasm from oocytes to embryos to identify the substance with PGC-inducing activity in model systems such as *Drosophila* ^65^, zebrafish ^66^, and *Xenopus* ^67^. To date, only a limited number of factors that can induce germ cells have been identified, and how the germ plasm assembles remains a key question in the field.

The molecular constituents of the germ plasm are best understood in *Drosophila* because of the powerful genetic screens which led to the identification of key factors of germline assembly and germ cell development ^68– 70^, including the germ plasm assembler, *oskar* ^71^. Alternative translation generates two forms of Oskar with distinct activities ^72^. The short form of Oskar is required for germ plasm assembly and function, whereas long Oskar lacks the assembly activity and instead is required to anchor germ plasm ^72– 74^. Until recently, Oskar (Osk) was viewed as a scaffolding protein that gathered germ plasm via interactions with a myriad of partners ^25, 74– 94^. The regions of Oskar mediating its association with partners such as Vasa defined Oskar functional domains ^73^. The recently elucidated Oskar crystal structure revealed that both Oskar dimerization and interaction with Vasa are mediated via the Osk N-terminal LOTUS domain, named after Limkain, Oskar and Tudor domain-containing proteins 5 and 7 ^83^. LOTUS is a globular domain present in several germ plasm/granule components ^95^, including the conserved Tudor family, first discovered for Tudor’s function in germ plasm assembly in *Drosophila* ^68^. This finding is in contrast to previous work that mapped the Vasa interaction to Oskar’s C-terminus ^73^. In the new study, Vasa-Oskar interactions were tested without RNAs to exclude RNA-mediated association and this may explain the different binding sites. These findings further support interaction between Vasa and Oskar and raise new questions and models to explain how these different Oskar complexes promote germ plasm formation and activity.

Another exciting aspect of the recent study is the evidence that short, but not long, Oskar associates directly with RNA. Surprisingly, this binding interaction is not via a canonical RNA-binding motif, but instead through a domain that resembles an enzymatically inactive SGNH hydrolase domain ^83^. SGNH hydrolases are a large enzyme family with thousands of members that are found in all life forms. An interesting property among some bacterial SGNH domain-containing proteins that may be of relevance to germ plasm assembly is their propensity to oligomerize to form amorphous aggregates or amyloid-like fibrils ^96, 97^. In addition, proteins encoded by some LINE (long interspersed nuclear elements) mobile genetic elements contain SGNH-like domains and mediate RNP assembly ^98, 99^. For example, the zebrafish LINE protein ZfL2-1 ORF1p forms multimers, binds nucleotides, and like Oskar possesses an SGNH-like domain that lacks overt RNA-binding domain structure ^98^. In addition, ORF1ps, including ZfL2-1, have been shown to function as chaperones. In this context, ORF1ps interact with the LINE RNA and are postulated to mediate rearrangement of the RNA into a stable conformation that protects the RNA from degradation, but later can be reversed to facilitate reverse transcription ^98^. Based on *in vitro* structure function studies, ZfL2-1 is postulated to mediate RNP assembly via interactions with positively charged peptides that bind RNA structural elements ^99^. It remains to be determined whether Oskar has similar chaperone functions, but it is easy to imagine how such an activity could apply to germ plasm RNAs, which must be translationally silent and protected from degradation during transport but later are translated in a specific ^96^ subcellular location. The unique functional domains and distinct interaction properties of Oskar isoforms discussed above provide new models to test the mechanism by which Oskar could promote RNP and germ plasm assembly either directly via its RNA-binding domain or directly or indirectly via its disordered LOTUS and interaction with Vasa.

Vertebrates, even those that use maternal inheritance to specify their germline, lack *oskar* and instead have a vertebrate-specific gene called *bucky ball* ( *buc*) or *vegetally localized 1* ( *velo1*) ^4, 100^. Buc shares features with Oskar in that both are localized in oocytes as RNAs and proteins ^4, 81, 101– 103^, both are required for and can organize germ plasm ^3, 4^, both have been viewed as unstructured proteins, both display complex post-transcriptional regulation at the level of splicing ^81, 104^ and RNA localization ^72, 81, 105^, and both have properties of self-assembling aggregates and interact with RNA-binding proteins and other factors at the RNA and protein levels ^73, 75– 90, 92, 94^. Whether or not Buc protein, like Osk, can directly bind RNAs is unknown. Based on homology searches, Buc lacks identifiable functional domains and thus the mechanisms by which it promotes Balbiani body formation, germ plasm assembly, and oocyte axis specification are not clear and have relied on identifying Buc interaction partners and mapping their interaction sites on Buc ^78, 81, 106^. However, Bucky ball and Oskar germ plasm factors also have some differences ( Figure 1). Specifically, Buc protein is present in early-stage oocytes ^81, 101^, whereas Osk protein is detected in late-stage oocytes ^72^, and the few reported Buc interaction partners are vertebrate-specific ^78^. Finally, *osk* RNA has functions independent of its protein-coding role ^84, 107^, whereas no evidence of protein-independent RNA functions of *buc* have been reported. These differences support convergent evolution or co-option of these genes as germ plasm assemblers; however, further analysis, including cross-species comparisons and rescue experiments, are required to determine the extent to which the activities of these germ plasm assemblers overlap.

Much less is understood about the molecular regulators of maternal germ plasm specification in vertebrates; however, recently, endogenous Buc protein was shown to localize to the cleavage furrows of early embryos ^101, 106^ by a maternal Kinesin 1 (Mkif5Ba)-dependent mechanism ^106^. Moreover, the ability of Buc to induce ectopic PGCs requires Mkif5Ba and Buc protein localization to the cleavage furrows ^106^. Similarly, formation of germ plasm aggregates in *Xenopus* requires the Kinesin-like protein Xklp2 ^108^, indicating a conserved role for microtubule motor-dependent assembly of germ plasm in these vertebrates. Because germ plasm is present in zebrafish M *kif5Ba* mutant embryos but germ cells are absent, this mutant provides evidence that inheritance of maternal germ plasm from oocytes alone is not sufficient to specify PGCs. Moreover, germline establishment requires proper spatiotemporal localization of PGC determinants.

## Germ plasm, germ granules, and germline identity

**Figure 2.**
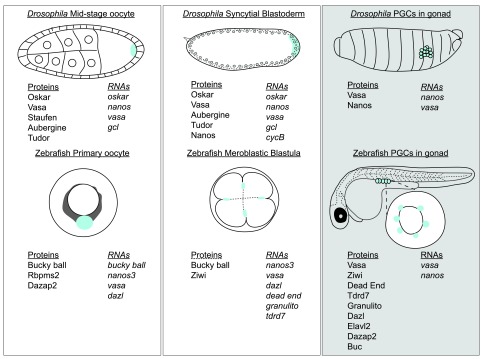
Comparison of maternal germ plasm and zygotic germ granule components between two organisms, *Drosophila* and zebrafish, that use maternal inheritance to specify their primordial germ cells (PGCs). The first column depicts the localization of germ plasm components (listed beneath the cartoon) at the posterior pole of mid-stage oocytes of *Drosophila* (top) and in the Balbiani body of early-stage oocytes of zebrafish. After fertilization, maternal germ plasm components (listed beneath the schematic) localize to specific membranes within the syncitial blastoderm of *Drosophila* and meroblastic cleavage (cells are incompletely separated and connected to the yolk) stage zebrafish embryos. The cells that receive these membranes develop as the PGCs. White rectangles depict maternal germ plasm components. The last column (grey rectangle) depicts PGCs of *Drosophila* and zebrafish after their migration to the gonad and indicates components of embryonic PGCs and germ granules. Dazap2, deleted in azoospermia-associated protein 2; Dazl, deleted in azoospermia-like; Elav, HuC; *gcl*, germ cell-less; *Rbpms2*, ribonuclear-binding protein with multiple splice isoforms 2.

The capacity of germ plasm to specify PGC fate has clear support from studies in *Drosophila* ^65^, zebrafish ^66^, and *Xenopus* ^67^, in which transplantation of the germ plasm can induce PGCs. In contrast, when germ granules are not properly segregated in *Caenorhabditis elegans* mutants, excess PGCs do not form nor do all cells adopt germline fates; instead, the cells take somatic fates ^109^. This example suggests that germ granules are not sufficient for PGC fate. These differences in germ cell induction capacity between granules and plasm raise the question of whether germ plasm and granules are based on their shared enrichment of RNA-binding proteins (RNAbps), RNAs, and other overlapping component ^4, 73, 78, 101, 106, 110^ manifestations of the same mechanism/structure operating at different stages or instead are distinct entities with unique properties. Alternatively, not all granules are equivalent. The maternal germ plasm associated with the maternal inheritance specification mode is continuous from oocyte to embryo, whereas perinuclear germ granules assemble only in PGCs, even in zygotically induced/discontinuous modes of germline specification. Thus, maybe these germline entities are not functionally equivalent. If this is the case, germ plasm from oocytes should contain germ cell-inducing factors, which germ granules of PGCs lack. Consistent with this notion, the perinuclear nuage in *Drosophila* oocytes is distinct from the polar granules nucleated by Oskar ^111^ during specification of pole cells/PGCs ^65^. Moreover, recent studies in *Drosophila*, *C. elegans*, and zebrafish provide evidence that germ granules are heterogeneous. In *Drosophila*, single RNA fluorescent *in situ* hybridization (FISH) analyses of granules revealed their organized architecture of “core” germ plasm proteins (e.g., Vasa and Oskar) distributed throughout granules and specific RNAs spatially arranged within granules ^112^. Significantly, although Oskar protein is present in granules, *oskar* RNA is not ^112, 113^ ( Figure 2). When chimeric *osk* with a *nanos* 3’ untranslated region (UTR) is directed to granules, germ cell numbers and Vasa expression are reduced ^113^, indicating that *osk* segregation from granules is needed for normal PGC development. Taken together, these findings are consistent with plasm and granules being distinct entities in *Drosophila*. Similarly, endogenous Buc protein localizes to germ plasm of oocytes and early embryos but is not detected in germ granules of PGCs ^106^. Moreover, *buc* RNA is a component of germ plasm of oocytes but not embryos, and even when overexpressed, Buc protein, but not *buc* RNA, localizes to germ granules of zebrafish PGCs ^4, 101, 106^ ( Figure 2). In addition, in zebrafish, *vasa* RNA but not Vasa protein is a component of the Balbiani body, where germ plasm localizes in oocytes ^18^. In contrast, Vasa protein is a PGC granule component in zebrafish ^11, 18, 114^ and other animals examined (reviewed in 15, 115, 116). In zebrafish, as in *Drosophila*, analysis of MS2-tagged and endogenous *vasa* and *nanos* RNAs indicates that granules are not equivalent ^117, 118^; therefore, it is likely that some but not all have the capacity to induce the germ cell fate or that these granules have another function.

In addition to their varied composition discussed above, other evidence indicates that germ plasm and germ granules may not be functionally equivalent. For example, the germ plasm and germ granules occupy distinct subcellar locations. In zebrafish and *Xenopus*, germ plasm associates with the endoplasmic reticulum in oocytes ^28^. In zebrafish embryos, germ plasm first accumulates at furrows of cleavage-stage embryos by a mechanism that involves RNA recruitment and clearance ^106, 119^, whereas germ granules are perinuclear and form after genome activation ^120^. In addition, the period when cells are competent to develop as PGCs, regardless of specification mode, precedes granule assembly and is developmentally restricted and brief, indicating that germ cell-inducing activity is tightly regulated. In *Drosophila*, rescue of *osk* mutants and the number of excess PGCs in overexpression contexts depend on Osk levels ^3, 71^. In zebrafish, expression of exogenous Buc after fertilization produces only a few additional PGCs, indicating that levels or timing may be limiting ^4, 106^. Similarly, in mice, high levels of the zinc finger transcriptional protein Blimp/Prdm1 are required to drive the epigenetic and cellular features of germ cells *in vivo* ^121^, and BLIMP1 specifies hPGCLCs in a similar dosage-dependent manner ^122^. These observations suggest that a limiting threshold or additional non-mutually exclusive factors (molecular, spatial, or temporal), or both, are essential for PGC identity. Accordingly, splitting determinants among sister cells, as occurs in *C. elegans* maternal-effect sterile ( *mes-1*) mutants ^109, 123^, would produce two cells lacking sufficient factors for germline fate. Notably, granules are also heterogeneous and dynamic structures in *C. elegans* ^124, 125^. Finally, other evidence from *C. elegans* shows that specific germ granule factors, including PGL-1 and PGL-3, induce aggregate/granule formation in non-germline cells without converting those cells to PGCs, further indicating that granules alone are not sufficient for germ cell identity/specification ^126, 127^.

Germ granules are enriched for RNA-binding proteins and proteins with other roles in post-transcriptional regulation; therefore, if germ granules do impart germline identity, it is possible that post-transcriptional regulation of other factors that may or may not be granule components is involved. If so, granules could impart germline fate only to cells that already express that factor; for example, Osk recruits components that regulate localization and translation of two germ granule components *nanos* and *pgc*, which promote patterning and pole cell development in flies ^110, 112, 128– 130^, and Buc must be localized to induce PGCs in zebrafish ^106^. Consistent with an essential role for granules in germ cells, depletion of one or more germ granule components in *C. elegans* causes sterility ^131– 133^. Recently, maternal *dazap2* was shown to maintain germ granules of zebrafish PGCs by acting epistatic to Tudor-7 and antagonistic to Dynein activity ^78^. Because PGCs are specified, and granules form but later are not maintained in PGCs lacking maternal Dazap2, M *dazap2* mutant germ cells provide an opportunity to explore potential roles of granules in maintenance of vertebrate PGCs. The historical view of nuage, germ plasm, and germ granules was that each of these entities would promote the germ cell fate beginning with their specification to maintenance of germline identity. Efforts to gain a deeper understanding of the components of germ granules and functional assessment of these conserved elements of PGCs have provided strong evidence for mechanisms to preserve or protect germline identity; therefore, it is worth considering the possibility that the germ granules of specified embryonic PGCs contribute to a mechanism that preserves germline identity rather than specification of PGC fate. Understanding the contribution of granules to germ cell development and fertility remains an active area of investigation.

## Germline specification in mammals

**Figure 3.**
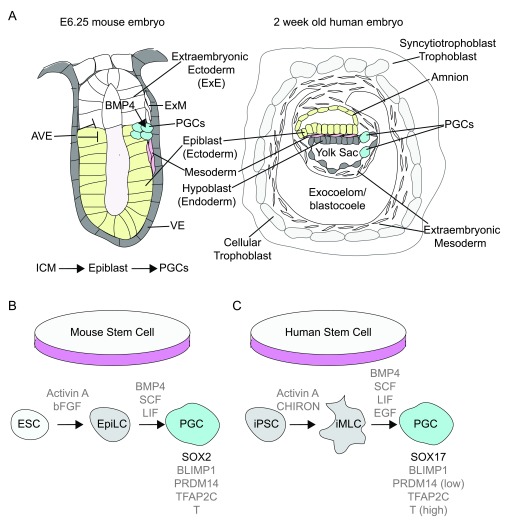
Primordial germ cell (PGC) induction in mammals. ( **A**) The early stages of PGC emergence in mouse and humans. In mouse, cues from extra-embryonic ectoderm, including bone morphogentic proteins (BMPs), induce transcriptional regulators that promote PGC identity in the cells of the adjacent posterior epiblast. In humans, PGCs are first detected around the onset of gastrulation within the endodermal yolk sac wall. The signals that induce human PGCs *in vivo* are not known. ( **B, C**) The molecular players involved in PGC specification from stem cells in culture. ( **B**) For mouse cells, the program to induce primordial germ cell-like cells (PGCLCs) from embryonic stem cells (ESCs) in culture requires transforming growth factor-beta (TGF-β) family members (Activin) and basic fibroblast growth factors (bFGFs) to promote an epiblast-like cell (EpiLC) state. Exposure of the EpiLCs to BMPs (BMP4) and stem cell factor (SCF) and leukemia inhibitory factor (LIF) converts the EpiLCs to cells with mouse PGC-specific gene expression profiles. ( **C**) For human cells, the program to induce PGCLCs from human-induced pluripotency cells (iPSCs) in culture like mouse requires TGF-β family members (Activin) and CHIRON to inhibit glycogen synthase kinase 3, an inhibitor of Wnt activity. This combination of factors promotes an induced mesoderm-like cell (iMLC) fate, which upon exposure to BMP4, LIF, SCF, plus epidermal growth factor (EGF) generates cells with human PGC-specific gene expression profiles. Notably, the transcription factor Sox2 is required in mouse cells, whereas Sox17 is necessary for human cells. In addition, PRDM14 is highly expressed in mouse PGCs and has been reported to be low or not expressed in human PGCs. AVE, anterior visceral endoderm; ExM, extra-embryonic mesoderm; ICM, inner cell mass; VE, visceral endoderm.

In mammals, the germ cells are not specified by inheritance of maternal cytoplasm (germ plasm), but instead are specified later by inductive signals. In the mouse embryo just before gastrulation, signals from extra-embryonic ectoderm and visceral endoderm are necessary to specify germ cells within the posterior epiblast that is adjacent to the forming primitive streak ( Figure 3). Unlike in flies and fish, in mice, no single factor that is necessary and sufficient to specify germ cells has been discovered. The earliest markers of mouse PGCs, including Blimp1 (Prdm1) and Prdm14, two critical factors that suppress somatic gene expression (thus promoting germ cell-specific gene programs) ^133– 137^ and developmental pluripotency-associated 3 (DPP3/Stella), are required to maintain rather than specify the PGCs ^138^. Strikingly, nearly all cells of the mouse pregastrula epiblast can express Prdm1 or Prdm14 when induced by ubiquitous expression of the bone morphogenetic protein ligands Bmp4 ^139^ and Bmp8b ^140^. Importantly, induced cells in culture can reconstitute functional sperm to germ cell-depleted neonates ^141^. However, *in vivo*, only a few cells within the posterior epiblast that are positioned proximal to the extra-embryonic ectoderm become PGCs ^142^. A Wnt3 signal from the epiblast primes these cells to respond to BMP produced by the extra-embryonic ectoderm ^141^. The mechanisms that limit germ cell induction in anterior regions are not fully understood. However, PGC formation involves anterior endoderm factors and additional BMP family members that repress Blimp1 and thus promote acquisition of pluripotency and PGC development specifically in the posterior epiblast ^141^. Other factors, such as microRNAs and their antagonists, contribute to PGC development (for example, by regulating Prdm1 expression) ^143^. However, the functions of these molecules are not confined to PGCs and so they do not represent germline determinants. Significant unresolved questions are how and what factors limit selection of just a few cells within this region to become PGCs. Moreover, the identity of the factor or factors that specify them remains unknown.

PGCs in humans, as in mice, are specified in extragonadal regions around the time of gastrulation onset. However, there are important differences in the timing and development of extra-embryonic tissues and likely PGC specification between human and mouse. During the second week of development, the human embryo is composed of epiblast and primitive endoderm, which gives rise to the yolk sac ( Figure 3). Owing to technical and ethical boundaries, lineage tracing to capture the first emergence of human PGCs prior to gastrulation has not been feasible. Nonetheless, human PGCs were identified in extragonadal regions more than 100 years ago and have been detected in human embryos in the Carnegie collection as early as stage 6 (around 2-week-old embryos) in the yolk sac endoderm in the vicinity of the developing allantois, an amniote structure involved in nutrition and waste removal ( Figure 3) ^144^. Functional studies of the earliest stages of PGC specification in humans would require manipulation and analysis of embryos within the first month following fertilization and this is not feasible. As an alternative to *in vivo* mammalian contexts, more tractable *in vitro* systems to study specification of mouse and human PGCs have been sought. In mice, key advances in PGC reprogramming paradigms have been facilitated by the development of germline reporters to identify and select for the lineage, including a mouse Vasa homolog (MVH) transgenic embryonic stem (ES) cell line, wherein the MVH promoter drives GFP reporter expression ^145^ and a germ cell-specific gcOct4-GFP ^146^. These tools enabled identification of germ cell-like cells in culture within just a few days. With markers in hand and using the germline-promoting factors defined from developmental studies, it was not long before mouse sperm ^147^ and egg ^146^ cells were derived from long-term ES cells. As germline stem cells (GSCs), PGCs are expected to generate cells that ultimately develop as functional gametes, sperm, or eggs that can produce a normal healthy fertile animal. The first animals generated from PGC-like cells (PGCLCs) were abnormal ^148^, a limitation to using such cells to study normal germline development or infertility. However, in 2011, healthy offspring were produced from male-derived PGCLCs building on the observation that nearly all of the pregastrula cells in the mouse could express key PGC factors Blimp1 (Prdm1) and Prdm14 in response to BMP4 ^149^. In that study, the authors recapitulated gametogenesis *in vitro* by first reprogramming cells to a pregastrula epiblast state before exposure to germ cell-differentiation cues ( Figure 3A,B). Later, the same group generated female PGCLCs with meiotic potential in reconstituted ovaries that produced fertile progeny after *in vitro* fertilization ^150^, indicating that the *in vitro* produced cells could develop as functional female gametes in the correct environment.

Encouraged by the success in establishing *in vitro* models of mouse PGC generation, several groups pursued *in vitro* models of human PGC development. With the mouse methods based on developmental paradigms in hand, the opportunity to discover the molecular programs responsible for producing the human germline seemed imminent. However, initial attempts to derive human PGCs quickly revealed that the mouse programming strategies were not effective, suggesting that despite conserved germ cell factors there must be differences in their mechanisms to generate germline cells ( Figure 3A). Thus, several groups sought and recently reported conditions to efficiently generate hPGCLCs ^8, 122, 151^ ( Figure 3C). Similar transcriptional programs associated with successful generation of hPGCLCs were discovered in two independent studies; one study used transcription activator-like effector nucleases to engineer human cell lines that express fluorescent reporters for BLIMP1 and TFAP2C to select for germ cells ^122^, and the second ^8^ imparted pluripotency by using a combination of four inhibitors and selected for germline cells by using a reporter for the conserved PGC-specific protein Nanos ^62, 63, 152– 154^. Interestingly, these studies indicate that PGC specification is somewhat more direct in human cells compared with mouse cells. In human cells, Sox17 induces and is necessary to specify hPGCLCs, with Blimp1 acting downstream to promote PGC gene expression and repress expression of mesendodermal, neuronal, and epigenetic reprogramming genes as the cells differentiate into hPGCLCs ^8, 122^. In mice, ES cells first transition to an epiblast state, and Blimp1, but not Sox17 ^155, 156^, represses somatic programs in nascent mPGCLCs ^149^ ( Figure 3). In both, epigenetic reprogramming is associated with PGCLC differentiation. However, the observed overlap in transcriptional programs between the human and mouse PGCLCs was surprisingly limited ^122^, indicating distinct PGC specification programs between mouse and human despite their reliance on shared signaling molecules. Notably, the hPGCLCs generated so far resemble early hPGCs and thus provide an unprecedented opportunity to study the earliest events in hPGC specification. However, late-stage hPGCLCs have not been obtained on the basis of the absence of markers that are normally expressed after PGCs migrate to the gonad, indicating that further differentiation or refinement of the protocols is required to obtain and study development of these later stages. Recapitulating development from stem cell to mature germ cell remains a challenge and an important step to realize the full potential of hPGCLCs to provide insight into human diseases, including infertility with a genetic basis or environmental/toxicological basis, as well as developmental disorders.

Once specified, PGCs in flies, fish, and mammals, but not *C. elegans*, must travel from their extragonadal site of specification to the presumptive gonad where the PGCs will differentiate into sperm in males or oocytes in females (reviewed in 157, 158). Successful migration to the gonad anlagen is essential for further PGC development and survival. With PGC markers and molecular and genetic approaches, key steps in PGC migration and the underlying cell behaviors have been defined and have been comprehensively reviewed elsewhere (for detailed reviews, see 157, 158). The RNA-binding protein Nanos regulates PGC migration and survival in *Drosophila* ^63, 159^, *C. elegans* ^160^, and zebrafish ^62, 161^, and *nanos* orthologs have a conserved role from invertebrates to humans in GSC maintenance ^62– 64, 152, 159, 160, 162– 166^. Despite conserved requirements for *nanos* orthologs, the relevant RNA targets of *nanos* genes and downstream mechanisms promoting germ cell survival are not fully understood. In non-mammalian animals, GSCs persist into adulthood and continue to produce new gametes throughout the reproductive life of the animal. In mammals, GSCs maintain spermatogenesis throughout the lifetime of adult males, but whether or not female mammals have the capacity to generate new oocytes after birth has been highly controversial and an area of active study (reviewed in 167). Coming back to August Weismann’s words, “The importance of such a theory lies primarily in its suggestiveness, by which alone it becomes a step towards the ideal at which we aim, namely, the formulation of the true and complete theory” ^1, 2^. Clearly, the existence of GSCs, either naturally occurring in adult mammalian females or *in vitro* produced, has important therapeutic implications for the field of reproductive medicine; however, further development of tools and studies of the mechanisms to specify and maintain stem cell niches and ovarian reserve in diverse systems are essential for an improved understanding of germline development and reproductive health.
